# A holistic carrier-bound immobilization approach for unspecific peroxygenase

**DOI:** 10.3389/fchem.2022.985997

**Published:** 2022-08-30

**Authors:** Piera De Santis, Noémi Petrovai, Lars-Erik Meyer, Markus Hobisch, Selin Kara

**Affiliations:** ^1^ Biocatalysis and Bioprocessing Group, Department of Biological and Chemical Engineering, Aarhus University, Aarhus, Denmark; ^2^ Institute of Technical Chemistry, Leibniz University Hannover, Hannover, Germany

**Keywords:** biocatalysis, unspecific peroxygenase, immobilization techniques, enzyme stability, process intensification

## Abstract

Unspecific peroxygenases (UPOs) are among the most studied enzymes in the last decade and their well-deserved fame owes to the enzyme’s ability of catalyzing the regio- and stereospecific hydroxylation of non-activated C–H bonds at the only expense of H_2_O_2_. This leads to more direct routes for the synthesis of different chiral compounds as well as to easier oxyfunctionalization of complex molecules. Unfortunately, due to the high sensitivity towards the process conditions, UPOs’ application at industrial level has been hampered until now. However, this challenge can be overcome by enzyme immobilization, a valid strategy that has been proven to give several benefits. Within this article, we present three different immobilization procedures suitable for UPOs and two of them led to very promising results. The immobilized enzyme, indeed, shows longer stability and increased robustness to reaction conditions. The immobilized enzyme half-life time is 15-fold higher than for the free *Aae*UPO PaDa-I and no enzyme deactivation occurred when incubated in organic media for 120 h. Moreover, *Aae*UPO PaDa-I is proved to be recycled and reused up to 7 times when immobilized.

## Introduction

During the past 2 decades, biocatalysis has become a valid and extensively used approach for chemical synthesis. On the one hand, this strategy helps to overcome the weaknesses of some traditional chemical processes, while on the other hand, it enables the development of more environmentally friendly processes (Wu et al., 2021). In addition, protein and process engineering, computer modeling and computational studies have paved the way for the industrial use of enzymes nowadays. One of the main benefits of using enzymes as catalysts is related to their high stereo- and regio-selectivity, which is particularly useful in the case of selective oxidation and oxyfunctionalization of non-activated C–H, C–C-, or C=C-bonds (Hobisch et al., 2021). In fact, from the organic chemistry perspective, site-specific oxyfunctionalization reactions are difficult to obtain since they rely on a fragile equilibrium between reactivity and selectivity, which, conversely, can be easily reached with enzymes. Therefore, biocatalysis opens the way to the selective oxyfunctionalization of organic molecules which plays a key role in the industrial scenario. In fact, these reactions are essential for the synthesis of building blocks, fine chemicals, functionalized polymers, and pharmaceutical compounds (Chanysheva et al., 2018; Grogan 2021).

Among the different enzyme families able to catalyze these crucial reactions, unspecific peroxygenases (UPOs) drew attention due to their interesting characteristics. Even though UPOs are heme-thiolate enzymes like the well-known P450 monooxygenases, they are independent from the use of expensive electron donors like nicotinamide adenine dinucleotide phosphate (NADPH) and auxiliary flavoproteins. In fact, UPOs only rely on hydrogen peroxide (H_2_O_2_) as cosubstrate and oxidant agent, solving the crucial problem of cofactor recycling (Molina-Espeja et al., 2015). Furthermore, as highlighted by Ramirez-Escudero et al*.*, UPOs do not only show the catalytic activity of P450s, the classical heme-peroxidase, but also a chloroperoxidative one from *Caldariomyces fumago* (Ramirez-Escudero et al., 2018; Hofrichter et al., 2020). This hybrid activity earned them a position as the first member of a subclass of oxidoreductases (EC.1.11.2.1) (Ramirez-Escudero et al., 2018). In addition, UPOs have a vast selection of transformations and a wide substrate scope that ensures their adoption to produce several fine chemicals. Unspecific peroxygenases from *Agrocybe aegerita*, in particular, is one of the most studied UPOs (Hofrichter and Ullrich 2014; Dong et al., 2018).

**FIGURE 1:**
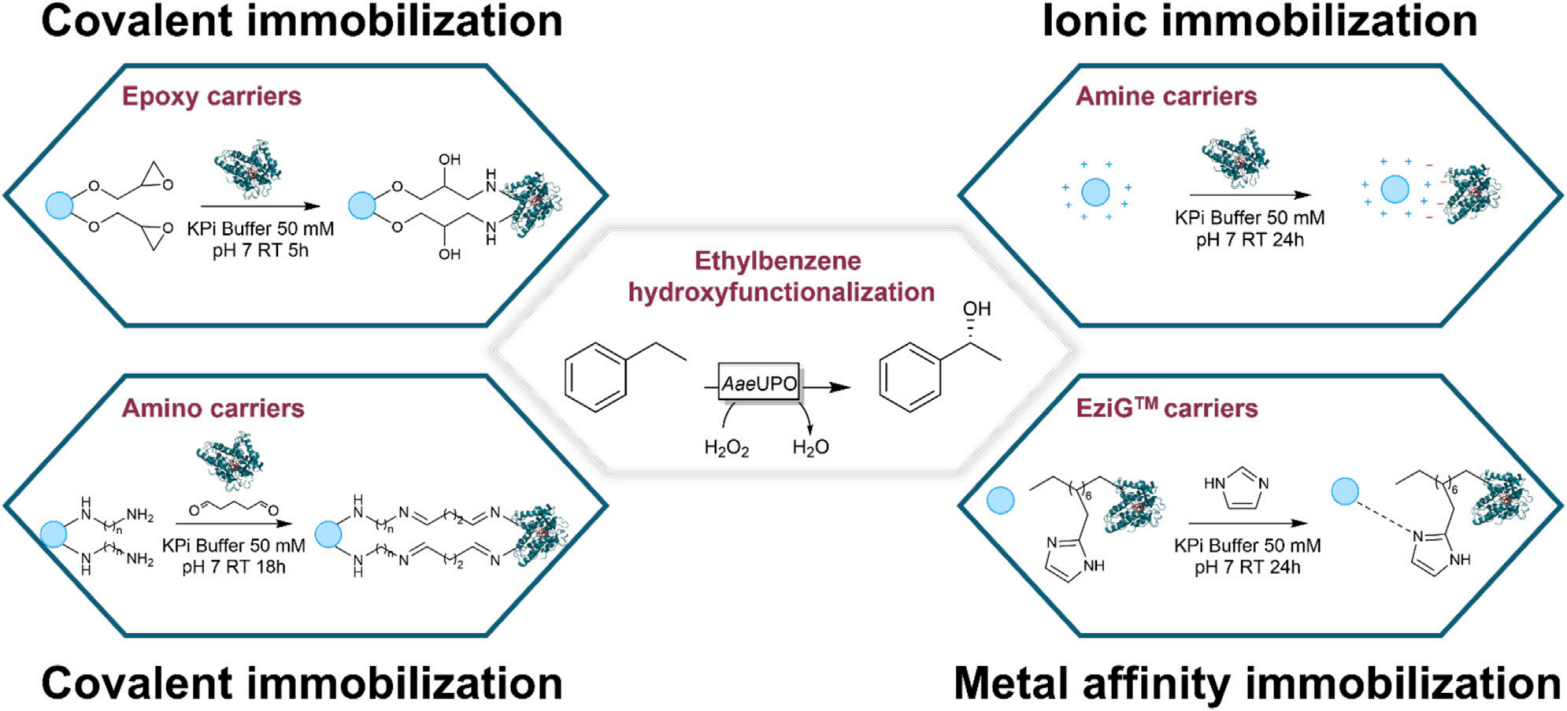
Schematic representation of the covalent, ionic, and metal affinity-binding immobilization techniques investigated in this study. The ethylbenzene hydroxy functionalization to (*R*)-1-phenylethanol is the model reaction chosen as proof-of-concept of immobilized enzyme applicability.

The bottleneck of UPOs’ application on industrial scale is nowadays due to the high cost of the heme-enzymes. Therefore, beside the improvement of the enzyme turnover number, which on the one hand was achieved *via* protein engineering (Molina-Espeja et al., 2014), and on the other hand by choosing the proper strategy for the *in situ* provision of the cosubstrate (Burek et al., 2019), another economically feasible approach to the use of UPOs is related to the enzyme recovery and recycling. The enzyme immobilization is the foremost strategy to do that (Figure 1).

As highlighted by Boudrant and coworkers, enzyme immobilization can represent the optimal solution to overcome different issues related to the use of biocatalysts (Boudrant et al., 2020). In fact, in this manner, is possible to improve the enzyme stability towards different organic solvents also at extreme values of pH and temperature (Sheldon, Basso et al., 2021). Higher biocatalyst loading into the reactors can be achieved, and, as aforementioned, this technique allows enzyme recyclability and simplified downstream processing (Thompson et al., 2019a). In addition, it also enables the use of enzymes in continuous processing (De Santis et al. 2020). Briefly, the enzyme immobilization leads to the development of a biocatalyst that can sustain the industrial requirements (higher stability and therewith higher overall productivity), and at the same time can be easily isolated and recovered from the reaction system.

This study offers an in-dept analysis of different carrier-bound immobilization methods to boost the use of UPOs at technical scales. In details, the experiments were performed with *Aae*UPO PaDa-I, a laboratory-evolved variant of UPO from *Agrocybe aegerita* benefitting from nine mutations including four in the signal peptide and five in the mature protein. Therewith, the secretion into the media and the stability in terms of temperature and cosolvents presence were significantly increased (Molina-Espeja et al., 2014; Molina-Espeja et al., 2015). So far, PaDa-I is the most promising mutant variant of UPOs.

We aimed to determine the most appropriate carrier bound immobilization protocol for the biocatalyst. The optimum (carrier) candidate should retain as much enzyme as possible and, at the same time, avoid any loss of its activity. Additionally, different parameters and carrier characteristics therefore need to be considered. As far as the latter is concerned, the size and the diameter of the pores play a crucial role. Parameters like (i) protein loading, (ii) immobilized enzyme activity, and (iii) immobilization yield and (iv) activity yield were used to evaluate the immobilization procedures (Liese and Hilterhaus 2013; Boudrant et al., 2020; Guisan et al., 2020).

## Materials and methods

General information. All solvents, reactants, and starting materials were received from commercial suppliers in the highest available purity (Sigma Aldrich, VWR, Carl Roth, Thermo Fisher) and used as received. Carriers for covalent immobilization (Lifetech™ ECR8285, ECR8215F, ECR8304F, ECR8404F, ECR8409F, ECR8315F, ECR8415F) as well as carriers for ionic immobilization (Lifetech™ ECR1508, ECR1604) were received from Purolite Life Sciences Ltd. (Llantrisant, United Kingdom) and treated as described below. Carriers for affinity immobilization of purified his-tagged enzyme (EziG™ Amber, Coral, and Opal) were received from EnginZyme A.B. (Solna, Sweden) and treated as described below. Potassium phosphate buffer (KPi buffer) was always freshly prepared in the lab for the experiments. All experiments were carried out under atmospheric conditions if not stated otherwise. For shaking and incubation of the carriers, a roller mixer from IKA (Staufen, Germany) was used. For photometric measurements, a temperature-controlled Cary 60 UV/Vis spectrophotometer from Agilent technologies (Santa Clara, California, United States) was used.

The mutant variant *Aae*UPO PaDa-I was used in the covalent and ionic immobilization procedures; conversely, the his-tagged PaDa-I was adopted for the experiments with the EziG™ carriers. Both the enzymes were produced *via* fermentation as described by Hobisch et al. (Hobisch and Kara 2021). The his-tagged PaDa-I was used as both crude preparation and purified enzyme; the purification was performed *via* immobilized-metal affinity chromatography (IMAC) with the Ni-NTA purification system and performed as described by the producer (see Supporting Information) (Fisher Scientific Thermo, 2015).

Covalent immobilization procedure. 1 g of each epoxy carrier (Lifetech™ ECR8285 and ECR8215F) was incubated in 2.5 mL of 50 mM KPi buffer (pH 7) on a roller mixer for 5 min followed by vacuum filtration for 5 min. This washing procedure was performed a total of three times. Subsequently, the supports were incubated in 3.0 ml of a PaDa-I dilution in 50 mM KPi buffer (pH 7, 0.27 mg_enzyme_ mL^−1^) for 5 h at room temperature under careful mixing and then for 14 h at 4°C. Finally, the carriers were vacuum filtered. The filtrate was washed again with 2.5 mL of 50 mM KPi buffer (pH 7) by mixing for 5 min and then decanting. This procedure was repeated one last time, and finally the support-immobilized PaDa-I was stored at 4 °C (Purolite Life Sciences 2015). All the three supernatants were stored at 4 °C for the further analyses (see below).

Amino carriers (Lifetech™ ECR8304F, ECR8404F, ECR8409F, ECR8315F and ECR8415F) were treated as described above but preactivated for 1 hour by adding 2.5 mL of a 25% (v/v) glutaraldehyde solution with stirring followed by filtration and washing (Purolite Life Sciences 2015).

Ionic immobilization procedure. 0.5 g of each amine carrier (Lifetech™ ECR1508 and ECR1604) was equilibrated with 2.5 mL of deionized water for 5 min on a roller mixer and then vacuum filtrated for 5 min. Afterwards, the resin was incubated in 1.7 mL of a PaDa-I dilution in 50 mM KPi buffer (pH 7, 0.24 mg_enzyme_ mL^−1^) for 24 h at room temperature under gentle mixing. Eventually, the carriers were vacuum filtered. The filtrate was washed again with 2.5 mL of 50 mM KPi buffer (pH 7) by mixing for 5 min and then decanting. This procedure was repeated one last time, and finally the support-immobilized PaDa-I was stored at 4 °C (Purolite Life Sciences 2015). All the three supernatants were stored at 4 °C for further analysis (see below).

Metal affinity binding immobilization procedure. 0.5 g of each carrier (EziG™ Amber, Coral, and Opal) was incubated with 2.9 mL of a PaDa-I dilution in 50 mM KPi buffer (pH 7, 1.4 mg_enzyme_ mL^−1^) for 24 h at room temperature under careful mixing. Finally, the carriers were vacuum filtered. The filtrate was washed again with 2.5 mL of 50 mM KPi buffer (pH 7) by mixing for 5 min and then decanting. This procedure was repeated one last time, and finally the support-immobilized PaDa-I was stored at 4 °C (Bormann et al., 2020). All the three supernatants were stored at 4 °C for the further analyses (see below).

Assessment of the enzyme immobilization. Bicinchoninic acid protein assay (Pierce™ 660 nm) was performed on all the wash fractions from the immobilization procedures (see above) to evaluate the mass of leached protein during the immobilization protocol. A calibration curve was obtained by measuring the absorbance of a dilution series of bovine serum albumin (BSA) in triplicates. By determining the concentration of the free PaDa-I in the wash fractions after the immobilization, the protein loading and immobilization yield were calculated (Eqss 1, 2, respectively) (Antharavally et al., 2009).

$$\mathrm{Protein loading}\left(\mathrm{PL}\right)\left(\frac{mg_{\mathrm{immo}.\mathrm{enzyme}}}{g_{\mathrm{wet carrier}}}\right)=\frac{m_{\mathrm{enzyme used initially}}-m_{\mathrm{wash}/\mathrm{filtrate fractions}}}{m_{\mathrm{wet carrier}}}$$ (1)

$$\mathrm{Immobilization yield}\left(\%\right)=\frac{m_{\mathrm{enzyme offered}}\;\left(mg_{\mathrm{enzyme}}\right)-m_{\mathrm{enzyme supernatants}}\;\left(mg_{\mathrm{enzyme}}\right)}{m_{\mathrm{enzyme offered}}\;\left(mg_{\mathrm{enzyme}}\right)}\times 100$$ (2)

To evaluate the immobilized enzyme’s activity, instead, a standard activity assay was performed on the immobilized enzyme as well as on the liquid fractions collected during the immobilization procedure. This assay is based on the absorbance of oxidized form of 2,2′-azino-bis(3-ethylbenzothiazoline-6-sulfonic acid) (ABTS) at 405 nm.

To investigate the liquid fraction, the PaDa-I-catalyzed oxidation of 990 μL 0.3 mM ABTS with 10 μL enzyme sample and 1.75 μL 3.5% (v/v) H_2_O_2_ was photometrically tracked over time and evaluated in triplicates in the time interval from 0.05 to 0.2 min; the measurements were performed in triplicates and the volumetric activity was calculated (Eq. 3) (Kadnikova and Kosti´ 2002).

Activityvol.(UmL)=ΔAbs405 nm×DF ×Vε405 nmABTS+°×v×d (3)

∆Abs_405 nm_ (min^−1^) = measured absorbance at 405 nm over time; DF (1) = dilution factor; d (cm) = light path; V (mL) = sample volume; v (mL) = enzyme volume in the sample; $\varepsilon_{405\;nm}^{ABTS^{+{}^{\circ}}}=\text{molar extinction coefficient}$ , 36.8 mM^−1^ cm^−1^ (Burek et al., 2019)

To investigate the immobilized enzyme, 10 mL of 0.3 mM ABTS, 1–10 mg of immobilized enzyme and 17.5 μL of 3.5% (v/v) H_2_O_2_ were stirred at 250 rpm at 25 °C. From the time of addition of the hydrogen peroxide, five samples (0.5 mL each) were taken every 30 s. The experiments were performed in triplicates and the activity was calculated Eq. 4.

Activityimmo.(Ugwet carrier)=ΔAbs405 nm×DF ×Vε405 nmABTS+°×mwet carrier×d (4)

By correlating the volumetric activity and the protein loading, the specific activity and the immobilization yield can be calculated Eqs 5, 6, respectively).

$$\mathrm{Activit}y_{\mathrm{spec}.}\left(\frac{U}{mg_{\mathrm{immo}.\mathrm{enzyme}}}\right)=\frac{A_{\mathrm{immo}}}{\mathrm{PL}}$$ (5)

$$\mathrm{Activity yield}\left(\%\right)=\frac{\mathrm{Activit}y_{\mathrm{spec}.}\left(\mathrm{Um}g_{\mathrm{immo}.\mathrm{enzyme}}^{-1}\right)\times m_{\mathrm{immo}.\mathrm{enzyme}}(mg_{\mathrm{enzyme})}}{m_{\mathrm{enzyme offered}}\left(mg_{\mathrm{enzyme}}\right)\times \mathrm{Activit}y_{\mathrm{spec}.}\left(\mathrm{Um}g_{\mathrm{free enzyme}}^{-1}\right)}\times 100$$ (6)

## Results and discussion

Three different immobilization techniques were investigated, each of which was based on a different type of interaction between the carrier and the enzyme (covalent, ionic, and metal affinity interactions, Figure 1). The first one relied on the formation of a covalent bond between the carrier functional groups and the enzyme amino acids. Two different carrier families were studied within the covalent binding technique: the epoxy and the amino supports, both supplied by Purolite Life Sciences (Llantrisant, United Kingdom).

As epoxy carriers, Lifetech™ ECR8285 and Lifetech™ ECR8215F were chosen and both resins are composed of polymethacrylate material functionalized with epoxy groups enabling a multipoint covalent attachment of the enzyme on the carrier surface. The differences between the two resins are their particle size and pore diameter (see Supporting Information). The formation of a covalent bond is here based on the interaction between nucleophilic amino acids (mainly lysines) and the epoxy group, which leads to the opening of the epoxydic ring (Figure 1) (Mateo et al., 2007). Because of the type of interaction, pH plays an important role for the success of the immobilization procedure. It is therefore important to emphasize that it is likely that an alkaline pH (instead of the neutral pH here applied) would have favored the nucleophilic attack and led to a higher immobilization yield. Unfortunately, this condition is not suitable for UPOs since activity is negatively affected by basic conditions (Molina-Espeja et al., 2014).

As amino carriers, Lifetech™ ECR8304F, ECR8404F, ECR8409F, ECR8315F and ECR8415F were selected. The differences between the five resins are their particle size, pore diameter, and the amino-spacer length (see Supporting Information). In contrast to the epoxy carrier case, the covalent bond is here obtained through a simple carbonyl-amine condensation reaction (see Figure 1). The resins need to be activated before via glutaraldehyde addition. Then, the activated carrier can bind the enzyme linking the second terminal aldehydes of glutaraldehyde and one of the amino groups of *Aae*UPO PaDa-I.

When it comes to the enzyme immobilization on amino carriers, not only different supports but also different enzyme-to-carrier ratios were investigated. Moreover, also the storage stability was determined up to 30 days after the immobilization.

For the characterization of the free and immobilized biocatalyst, the well-accepted assay system oxidizing ABTS to its single radical specie (ABTS^•+^) was used to determine enzyme activity (Kadnikova and Kosti´ 2002). Conversely, the protein loading was calculated according to the Pierce™ protein concentration assay. Both epoxy and amino supports showed a similar trend: higher protein loadings and activities were observed when supports with larger pore diameters were used. Moreover, comparison of the amino carriers also shows that shorter spacer lengths lead to higher protein loading and activity (Table 1).

**TABLE 1:** Screening results obtained for the covalent immobilization of *Aae*UPO PaDa-I.

Carriers^[a]^	Protein loading (mg_protein_/g_carrier_)	Immobilization yield (%)	Specific activity (U/mg)^[b]^	Activity yield (%)	Pore diameter (Å)	Spacer length^[^ ^c^ ^]^
*Epoxy carriers*						
ECR8285	0.250 ± 0.004	54	4.7	0.44	400–600	-
ECR8215F	0.484 ± 0.004	54	44 ± 4	4	1200–1800	-
*Amino carriers*						
ECR8304F	0.670 ± 0.007	57	2	0.2	300–600	Short spacer
ECR8404F	0.40 ± 0.09	32	n.d.^[d]^	n.d	300–600	Long spacer
ECR8409F	0.43 ± 0.01	35	n.d	n.d	600–1200	Long spacer
ECR8315F	0.616 ± 0.006	55	27.5 ± 0.5	3	1200–1800	Short spacer
ECR8415F	0.45 ± 0.05	28	21 ± 5	1	1200–1800	Long spacer

a All the here evaluated carriers are part of the Lifetech™^.^

ECR, resins supplied by Purolite Ltd. The resins are characterized by a common specific particle size of 150–300 μm, except for ECR8285 whom shows a particle size of 250–1000 µm.

b per mg of enzyme immobilized.

c Short spacer: C2, long spacer: C6.

d Not detected.

**FIGURE 2:**
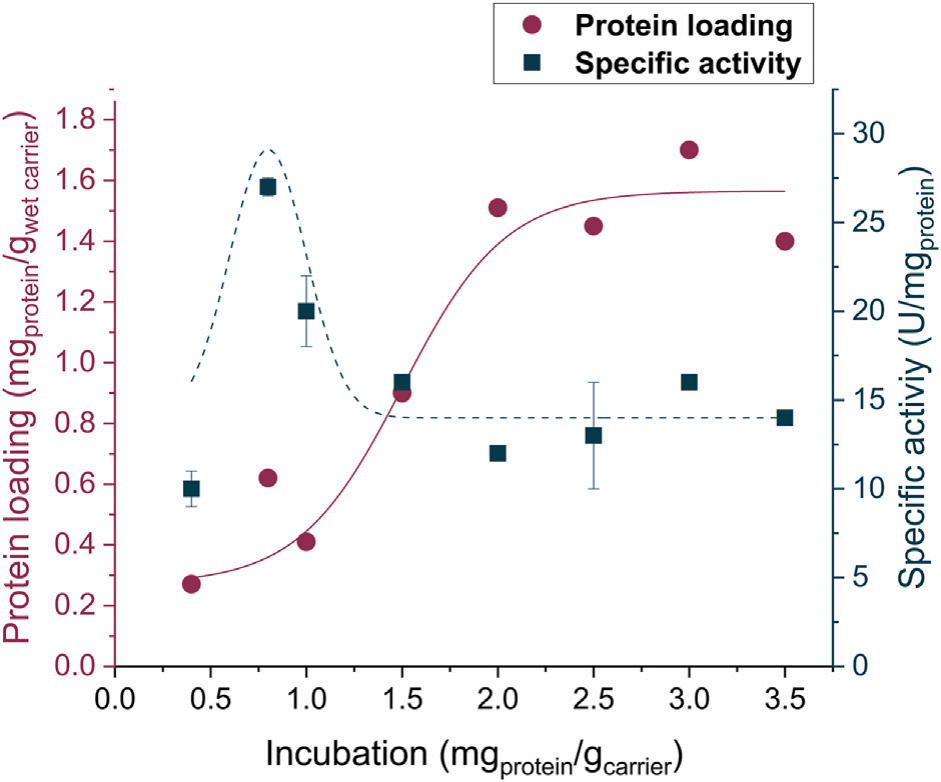
Activity and protein loading of the enzyme being immobilized on the amino carrier ECR8315F. The reported lines in the graph are only a guide to the eye. Experiments were performed in triplicates while for 1.5 and 2.0 mg_protein_/g_carrier_ values, the activity results showed up to 10-fold higher standard deviations.

Among the supports evaluated for covalent immobilization, Lifetech™ ECR8315F led to the highest protein loading and enzyme activity. Therefore, the immobilization procedure was further optimized for this carrier. Different enzyme-to-carrier ratios were evaluated (from 0.4 to 3.5 mg_protein_/g_carrier_) and, as highlighted in Figure 2, the 0.8 mg_protein_ g_carrier_^−1^ showed the right balance between a high protein loading and a satisfactory activity. It is worth noticing that by increasing the enzyme-to-carrier ratio, even if there is an increment in the protein loading, the enzyme activity tends to fluctuate or even decrease. This phenomenon might be attributed to the formation of multilayers on the carrier surface.

Additionally, the long-term process stability of immobilized *Aae*UPO PaDa-I was determined over 30 days and up to 60% of the enzyme activity was retained. Within the ionic immobilization strategy, two different resins from Purolite Life Sciences were evaluated (Lifetech™ ECR1508 and ECR1604) (see Supporting Information). Here, the carriers differ according to their functional group, a tertiary, and a quaternary amine (Table 2), and the enzyme is retained due to ionic interactions being established with the carrier’s and enzyme’s surface charge. Therefore, parameters like the isoelectric point of the enzyme, the optimal pH, and the ionic strength of the immobilization buffer must be carefully selected.

**TABLE 2:** Screening results obtained for the ionic immobilization of *Aae*UPO PaDa-I.

Carriers^[a]^	Protein loading (mg_protein_/g_carrier_)	Immobilization yield (%)	Specific activity (U/mg)^[b]^	Activity yield (%)
ECR1508	0.658 ± 0.002	38	9 ± 1	1
ECR1604	0.833 ± 0.003	61	7 ± 3	1

a All the here evaluated carriers are part of the Lifetech™ supplied by Purolite Ltd. The resins are characterized by a common specific particle size of 150–300 µm.

b Per mg of enzyme immobilized.

When *Aae*UPO PaDa-I is immobilized on Lifetech™ ECR1604 (quaternary amine), we observed a slightly higher protein loading (20%) compared to the enzyme being immobilized on Lifetech™ ECR1508 (tertiary amine). We hypothesize that the interaction between the negatively charged enzyme and the–NR_3_^+^ functional group is stronger compared to the–NR_2_ moiety. Conversely, the immobilized enzyme observes a similar activity when immobilized on both carriers (Table 2). Furthermore, the activity was measured 4 weeks after the immobilization and remained steady.

**FIGURE 3:**
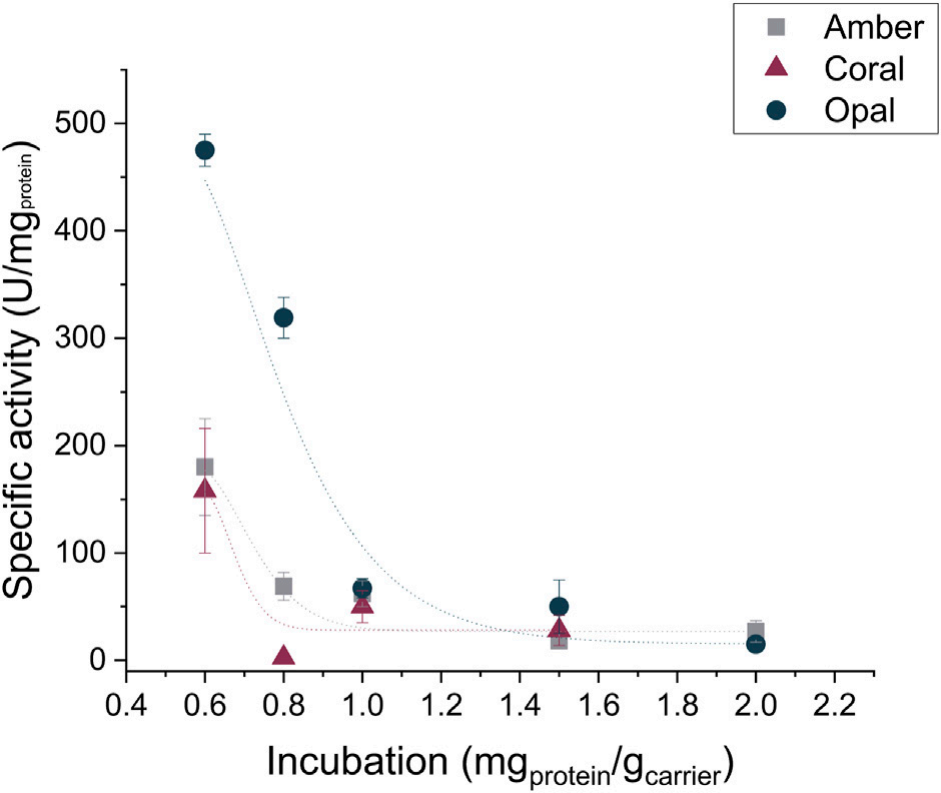
Activity of the enzyme being immobilized on the Amber, Coral, and Opal EziG™ carriers incubated at different protein-to-carrier ratios. The reported lines in the graph are only a guide to the eye.

As regards the metal affinity immobilization technique, we investigated the three porous carriers from EnginZyme A.B. (Solna, Sweden): EziG™ Amber, Coral, and Opal. These three carriers are made of a controlled-pore glass material covered by an organic polymer leading to hydrophobic, semi-hydrophobic, or hydrophilic resins (see Supporting Information). Additionally, Fe^3+^ ions are linked to the carrier surface. For the enzyme immobilization to occur, the cations interact with the lone pair of electrons of a histidine group (Cassimjee et al., 2018); therefore, his-tagged PaDa-I was used for the following immobilization investigations. When applied to purified his-tagged PaDa-I, we observed a significantly higher specific activity for immobilized enzyme on the Opal EziG™ carrier although the protein loading is similar for all carriers (Figure 3, Table 3).

**TABLE 3:** Screening results obtained for the affinity immobilization of purified his-tagged *Aae*UPO PaDa-I.

Carriers^[^ ^a^ ^]^ (optimum ratio)	Protein loading (mg_protein_/g_carrier_)	Immobilization yield (%)	Specific activity (U/mg)^[^ ^b^ ^]^	Activity yield (%)	Pore diameter (Å)
Amber (0.8)	0.37 ± 0.01	46	69 ± 13	11	300
Coral (1.0)	0.27 ± 0.03	45	50 ± 16	5	300
Opal (1.2)	0.76 ± 0.02	92	67 ± 9	55	500

a All the evaluated carriers are part of the EziG™ carriers supplied by EnginZyme A.B.; for each carrier, only the data related to the optimum enzyme-to-carrier ratio are reported in the table. The Amber one is a controlled pore glass material coated with a semi-hydrophobic polymer, the Coral is covered by a hydrophobic polymer, while the Opal carrier is made only of glass. In all cases, the particle size is 75–125 μm.

b Per mg of enzyme immobilized.

Again, we evaluated different enzyme-to-carrier ratios to reach the optimal protein loading without compromising the enzyme activity and found an optimal ratio at 0.8 mg_PaDa-I_ g_carrier_^−1^ for EziG™ Amber and 1.0 mg_PaDa-I_ g_carrier_^−1^ EziG™ Coral. Eventually, the highest specific activity was reached when the purified his tagged PaDa-I was immobilized on EziG™ Opal carrier with a final enzyme-to-carrier ratio of 1.2 mg_PaDa-I_ g_carrier_^−1^ (Supplementary Figure 3SI; Supplementary Tables S7–9). Moreover, the activity of the immobilized enzyme retained up to 1 month.

Further experiments were devoted to the proof-of-concept of our model reaction system, the hydroxy functionalization of ethylbenzene to the enantiopure (*R*)-1-phenylethanol. In this case, the covalently immobilized PaDa-I (Lifetech™ ECR8315F) was adopted and different parameters like (i) the amount of immobilized enzyme, (ii) the shaking intensity and (iii) the reaction temperature were optimized (see Supporting Information). These experiments were crucial to prove not only the applicability of the developed immobilization procedure to the model reaction, but also that the covalent enzyme immobilization do not affect either the regio- or the enantioselectivity properties of the enzyme. However, it cannot be omitted that also the (*R*)-1-phenylethanol overoxidation to acetophenone occurred as a side-reaction; when the optimum conditions are applied, a target product selectivity of 74% is achieved. Yet this datum was not an unforeseen phenomenon; in fact, even if the kinetics parameter regulating the alcohol overoxidation are still under debate, the ketone formation is a well-known side rection of UPOs catalysis (Kluge et al., 2012).

**FIGURE 4:**
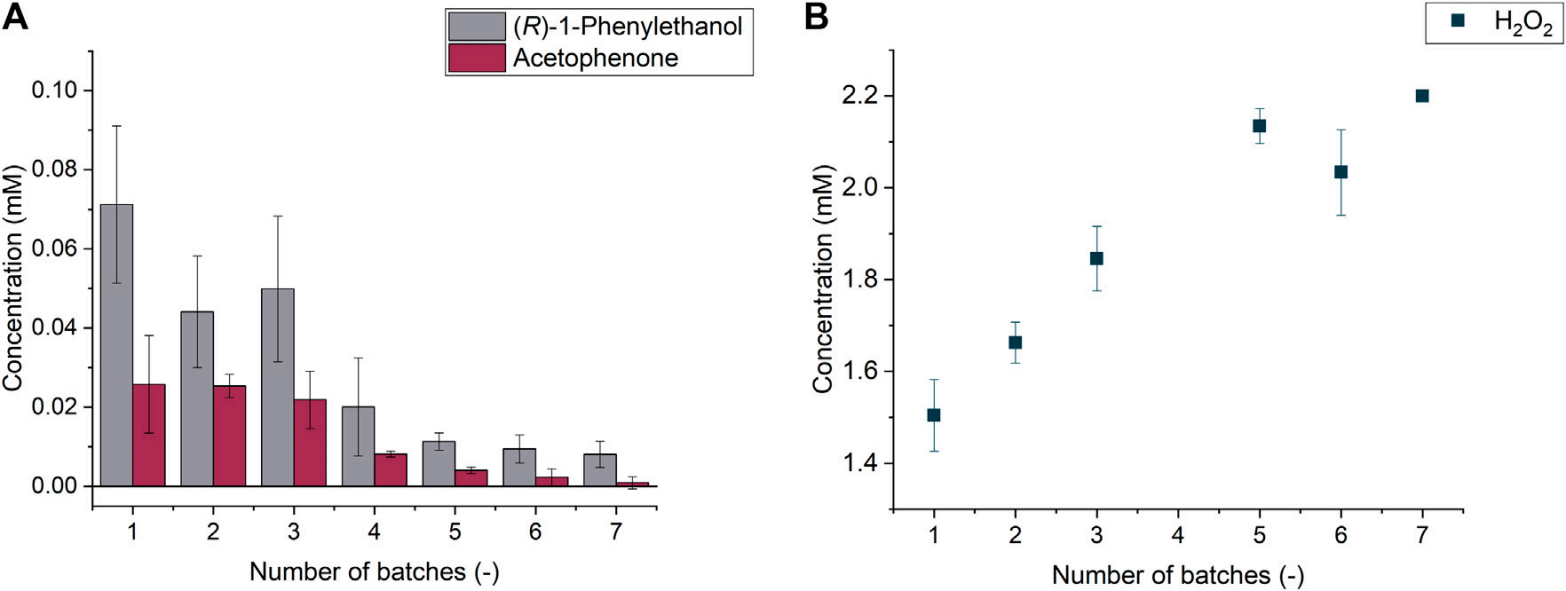
(*R*)-1-phenylethanol and acetophenone formation through the repeated batches **(A)** hydrogen peroxide concentration determined at the end of each batch **(B)**.

The optimized conditions laid the foundation for additional experiments about the enzyme recyclability (see Supporting Information). Repetitive batch experiments were indeed successfully conducted and demonstrated that the covalently immobilized enzyme can be recycle up to 7 times, with a loss of product formation of 89% after six batches (see Figure 4).

Before concluding this chapter, it is worth observing that the aforementioned procedures are only part of the numerous available immobilization techniques. Unexpectedly, despite the countless advantages of enzymatic immobilization as well as the big appeal UPOs have captured since their discovery in 2004, only few research about the combination of these two core topics have been published so far. For instance, one of the first published UPOs carrier bound immobilization procedures, involved the recombinant unspecific peroxygenase from *Agrocybe aegerita* (*rAae*UPO) on Relizyme™, which is a polymethacrylate resin functionalized with hexamethylenamino groups (Fernandez-Fueyo et al., 2016). Similar to the aforementioned study, also Fernandez-Fueyo and coworkers evaluated the enzyme immobilization efficiency via protein concentration assay of the supernatant and via ABTS activity assay. When comparing their results with the ones shown before (Table 1) a similar behavior can be noted: the loss of enzyme performance (in both cases around 20-fold) is well-balanced by the increase in the enzyme stability. Recently, the same enzyme was chosen by Carballares and coworkers to investigate the effect of the pH in ion exchange immobilization procedures on cationic supports; after an in-depth analysis, different activity assays proved that the best results are achieved with MANAE agarose (Carballares et al., 2021). As it also emerges from the experiments conducted (see Table 2), high immobilization yield value can be achieved via ionic immobilization. However, the pH control necessary to perform this immobilization can negatively affect the enzyme activity. In fact, as proved by Carballares et al.*,* albeit the ionic immobilization performed at pH 9 led to an immobilization yield of almost 100%, it also caused a decrease in enzyme activity and stability; according to these results they concluded that, to take the most advantage from the immobilization procedure, the *rAae*UPO needs to be immobilized at pH 5. Turning back to the covalent immobilization, Molina-Espeja et al*.* proved the feasibility of directed unique-point covalent immobilization (DUCI) strategy by synthetizing and immobilizing a new PaDa-I-Cys variant, through the formation of a single disulfide bridge between the just introduced cysteine and the carrier (Molina-Espeja et al., 2019). The main advantage of this immobilization procedure is the strict control on enzyme orientation exercised by the activated carrier. One of the two carrier materials studied for the directed unique-point enzyme immobilization, is a polymethacrylate epoxy activated carrier which led to results comparable to the ones presented for the Lifetech™ ECR8215F (see Table 1). Moreover, the authors used fluorescence confocal microscopy to visualize the enzyme bound to the epoxy-carrier and, since only a negligible amount of the non-modified PaDa-I was linked to the carriers, to prove the selectivity of the immobilization procedure.

Carrier-free immobilization strategies can also represent a valid alternative; carrier-free methods generally show advantages like high volumetric activity and stability against unnatural condition. According to authors’ knowledge, so far Poraj Kobielska and coworkers have demonstrated the feasibility of unspecific peroxygenase encapsulation in polyvinylalcohol/polyethylenglycol gel beads (Poraj-Kobielska et al., 2015) and Kara and coworkers in alginate beads (Hobisch et al., 2020).

Eventually, it is also worth to mention the positive effect that *in-silico* analysis can have in the development of an immobilization procedure. These can indeed act as guidelines helping us to shortlist the countless procedures, to point out the optimal conditions and to foresee the immobilized enzyme behavior. A tool like molecular docking, for example, can help better understanding the protein domains involved in the anchoring phase and predict the effect on the enzyme active site (Holyavka et al., 2016). Similarly, software like Swiss Pdb Viewer and Maestro identify the amino acid residues on the enzyme surface and provide information about their special position. Both were indeed used by Sakibaev and coworkers to determine the distance between the active center of the enzyme and the potential sites for binding, an important parameter to predict the effect of the immobilization procedure on the enzyme activity (Sakibaev et al., 2019). An alternative is represented by algorithms such as LIGRe (ligand interacting group reactivity) that determines the amino acids reactivity at a given pH highlighting the regions of the enzyme prone to interact with the carrier functional group (Torres-Salas, del Monte-Martinez et al., 2011). Moreover, microscopes such as scanning electron microscope (SEM) and fluorescence confocal microscopy are often used to display the enzyme distribution throughout the carrier pores and to predict substrate/product diffusion limitation problems within the support, respectively (Molina-Espeja et al., 2019; Ahmad et al., 2020).

## Conclusion

Among the different immobilization strategies investigated, metal affinity binding using the Opal EziG™ support, proved to be the most suitable method for *Aae*UPO PaDa-I. The optimized condition led to an immobilization yield of 92% and to an activity yield of 55%; moreover, the 75% of enzyme activity was kept up to 30 days. However, it is worth noting that these promising results were obtained using purified PaDa-I, which consists of an extra cost that negatively affect the labour and overhead (L&O) costs (Thompson et al., 2019b). Comparing only the results of the crude enzyme extract immobilization procedures, covalent immobilization with the amino carrier Lifetech™ ECR8315F is found to be the optimum. When immobilized on this carrier, the enzyme has an immobilization yield of 55% and an activity yield of 2%. Moreover, even if 40% of enzyme activity is lost after 30 days, the half-life time of the immobilized enzyme is 15-fold higher than for the free PaDa-I. Eventually, the covalently immobilized enzyme was adopted as catalyst in the ethylbenzene hydroxy-functionalization reaction (proof-of-concept) and to validate the hypothesis of enzyme recyclability (until 7 times). According to Thompson *et al.*, the obtained results respect industrial requirements like high enzyme loading (>10wt%) and partially satisfy requirements such as high retained activity (>50%) and enzyme recyclability (Thompson et al., 2019a). Moreover, an upscale from 1 to 10 g immobilization performed with the Lifetech™ ECR8315F carrier with no negative consequences, reveals its potential use on larger scales, e.g. in flow, and foreshadows that the system is robust enough for further increase. Considering this very promising results, future experiments will be devoted to further improve immobilization and reaction conditions in order to continue reducing the gap between the enzyme properties and the industrial requirements.

## Data Availability

The original contributions presented in the study are included in the article/Supplementary Material further inquiries can be directed to the corresponding author.
