# Supplementary material for: A holistic carrier-bound immobilization approach for unspecific peroxygenase
Source: Front Chem. 2022 Aug 30;10:985997. doi: 10.3389/fchem.2022.985997 (PMC9468545; doi:10.3389/fchem.2022.985997)
Supplement: Supplementary file 1 [file DataSheet1.PDF]

Supporting Information

A holistic carrier-bound immobilization approach for unspecific peroxygenase

Piera De Santis[1], Noémi Petrovai[1], Lars-Erik Meyer[1], Markus Hobisch[1], Selin Kara[1,2*]

[1] Biocatalysis and Bioprocessing Group, Department of Biological and Chemical Engineering, Aarhus University, Aarhus, Denmark
[2] Institute of Technical Chemistry, Leibniz University Hannover, Hannover, Germany

*** Correspondence:**
Selin Kara
selin.kara@bce.au.dk

TABLE OF CONTENTS

EXPERIMENTAL PROCEDURES 2

Chemicals 2

Enzymes production 2

Carrier materials 2

His-tagged PaDa-I purification 3

Electrophoresis analysis of protein fractions 3

Analysis of optimum enzyme-to-carrier ratio 3

Analysis of enzyme half-life time 4

Enzyme stability experiment 4

Enzyme leaching experiment 4

Progress curve analyses 4

Repetitive batch experiments 4

GC analysis 5

Hydrogen peroxide (H_2O_2) quantification 6

RESULTS 8

Protein purification 8

Electrophoresis analysis of protein fractions 8

Analysis of enzyme-to-carrier ratios 9

Analysis of enzyme half-life time determination 11

Enzyme stability experiment 11

Enzyme leaching experiment 11

Progress curves 12

Repetitive batch experiments 13

REFERENCES 13

EXPERIMENTAL PROCEDURES

Chemicals

All the chemicals were purchased from commercial suppliers in the highest available purity (Sigma Aldrich, VWR, Carl Roth, Thermo Fisher) and used as received.

Enzymes production

The laboratory evolved *Aae*UPO PaDa-I (plasmid kindly supplied by Professor Alcalde) and the his-tagged PaDa-I (plasmid kindly provided by DFI Frankfurt), both expressed in *Pichia pastoris*, were produced *via* fermentation as published by M. Hobisch et al..(Hobisch and Kara 2021)

Carrier materials

For the covalent immobilization the epoxy and amino carriers were supplied from Purolite Life Sciences (Llantrisant, United Kingdom).(Purolite Life Sciences 2015)

Table S1. *Properties of the carrier materials used for covalent enzyme immobilization.*

Carrier	Material	Functional group	Particle size (µm)	Pore diameter (Å)	Spacer length
**ECR8285**	Polymethacrylate	Epoxy	250-1000	450-650	-
**ECR8215F**	Polymethacrylate	Epoxy/butyl	150-300	1200-1800	-
**ECR8304F**	Polymethacrylate	Amino	150-300	300-600	C2
**ECR8404F**	Polymethacrylate	Amino	150-300	300-600	C6
**ECR8409F**	Polymethacrylate	Amino	150-300	600-1200	C6
**ECR8315F**	Polymethacrylate	Amino	150-300	1200-1800	C2
**ECR8415F**	Polymethacrylate	Amino	150-300	1200-1800	C6

For the ionic immobilization the carriers were supplied from Purolite Life Sciences (Llantrisant, United Kingdom).

Table S2. *Properties of the carrier materials used for ionic enzyme immobilization.*

Carrier	Material	Functional group	Particle size (µm)	Pore diameter (Å)
**ECR1508**	Polystyrene	$-NR_2$	300-1200	n.a.
**ECR1604**	Polystyrene	$-NR_3^+Cl^-$	300-1200	n.a.

n.a. (not available)

For the metal affinity binding the three porous carriers from EnginZyme (Solna, Sweden): EziG^{TM} Amber, Coral and Opal were applied(Cassimjee and Federsel 2018).

Table S3. Properties of the carrier materials used for metal affinity binding.

Carrier	Material	Functional group	Particle size (µm)	Pore diameter (Å)
**Amber**	Controlled porosity glass	Hydrophobic organic polymer	n.a.	300
**Coral**	Controlled porosity glass	Semi-hydrophilic organic polymer	n.a.	300
**Opal**	Controlled porosity glass	Hydrophilic	n.a.	500

n.a. (not available)

His-tagged PaDa-I purification

The his-tagged PaDa-I was purified *via* metal affinity chromatography (IMAC). This procedure promotes the separation between the his-tagged enzyme and all the other components of the crude enzyme concentrate that can interfere with the enzyme immobilization.

The purification was performed by packing a column with Ni-NTA resin (5 mL, from now on known as column volume, CV) from Thermo Fisher Scientific according to the producer's.(Thermo Fisher Scientific 2015) The resin was washed with 3 CV of distilled water and 5 CV of washing buffer. Meanwhile, the crude enzyme concentrate (5 mL) was filtered with a 0.22 µm syringe filter and loaded onto the column and 5 mL of elute were collected as "outflow". Then, 10 CV of washing buffer were added to the column and the first 15 mL of elute were collected as "wash buffer". During these steps, all the proteins without the his-tag were washed away from the column. To collect the purified protein, 3 CV of elution buffer were added; each of them was collected in a different falcon tube, labelled as 1st, 2nd and 3rd elute, respectively.

After the purification procedure, the purified his-PaDa-I needed to be desalted and a 10 kDa Amicon® Ultra-15 Centrifugal Filter Units was used. The enzyme solution was centrifuged at 4000 rpm and 4 °C for 20 minutes; the sample volume was reduced from 5 to 1 mL. After that, 6 mL of the desalting buffer were added to the centrifugal filter and the sample was centrifuged again; this step was done twice. Eventually, the purified his-PaDa-I was collected and stored at 4 °C.

Electrophoresis analysis of protein fractions

After its purification, the enzyme was analyzed *via* sodium dodecyl sulphate-polyacrylamide Gel Electrophoresis (SDS PAGE). The purified enzyme (30 µL) was mixed with 10 µL of loading buffer 4X[1] and then heated to 90 °C for 10 minutes. Afterwards, the samples and the protein marker ladder were loaded onto the gel, which was placed in an electrophoretic chamber and 140 V were applied to the system for 1 hour and 15 minutes. Coomassie Brilliant Blue dye was used to stain the gel and to visualize the proteins.

Analysis of optimum enzyme-to-carrier ratio

To optimize the covalent immobilization procedure on the amino carrier ECR8315F and the metal affinity binding one on the EziG™ carriers, different enzyme-to-carrier ratios were investigated. The carrier materials were incubated, as described in the Materials and Methods section of the article with increasing amount of free PaDa-I with the aim of determining the optimum amount of enzyme that

[1] Loading buffer composition: Tris-HCl pH 6.8, SDS, bromophenol blue glycerol and β-mercaptoethanol.

can be immobilized ensuring a good protein loading without compromising neither the enzyme activity nor causing enzyme leaching.

Analysis of enzyme half-life time

The half-life time ($t_{½}$) of both the free and covalently immobilized AaeUPO PaDa-I was determined by measuring the enzyme activity via ABTS assay at regular time intervals for up to one month; in between the samples were stored at 4 °C in plastic vials.

Enzyme stability experiment

A stability experiment was performed to assess the influence of organic solvents on the immobilized enzyme activity. The immobilized enzyme (ca. 15 mg) was incubated in a glass vial with 1.6 mL of ethylbenzene (pure and 1 mM in KPi buffer 50 mM pH 7), (*R*)-1-phenyl ethanol (pure and 1 mM in KPi buffer 50 mM pH 7) and acetophenone (pure and 1 mM in KPi buffer 50mM pH 7). The immobilized enzyme was collected regularly (up to 120 hours), washed in KPi buffer for 5 mins and then, the immobilized enzyme activity was tested *via* ABTS assay (as described in the Materials and Methods section of the article).

Enzyme leaching experiment

The final aim of the experiment was to determine if any enzyme leaching from the carrier material would have occurred when the enzyme is incubated in conditions that mimic the reaction environment. The immobilized enzyme (ca. 15 mg) was incubated in a glass vial with 1.6 mL of ethylbenzene (pure and 1 mM in KPi buffer 50mM pH 7), (*R*)-1-phenyl ethanol (pure and 1 mM in KPi buffer 50mM pH 7) and acetophenone (pure and 1 mM in KPi buffer 50mM pH 7). Samples were collected regularly (up to 120 hours) and analyzed *via* Pierce protein concentration assay (as described in the Materials and Methods section of the article).

Progress curve analyses

The reaction in aqueous media was conducted with ethylbenzene as a model substrate. Due to the solubility issue of ethylbenzene (less than 1.4 mM at 20 °C), the enzyme immobilized on amino carrier ECR8315F was incubated with 1.6 mL of 1 mM ethylbenzene in KPi buffer 50 mM pH 7 and 3.1 µL of 3.5% H_2O_2 v/v. The reaction occurred in a 2 mL glass vial placed in a thermo-shaker (Hettich Lab Technology, Netherlands) at 25 °C and 250 rpm.

Different properties such as the amount of immobilized enzyme, rpm and temperature were tuned with the aim of optimizing the product formation and selectivity. Therefore, several experiments were conducted varying one parameter at a time; samples were collected regularly (up to 60 minutes) and analyzed *via* GC-FID analysis.

Repetitive batch experiments

The immobilized enzyme recyclability was ascertained *via* a repeated batch experiment. The enzyme immobilized on amino carrier ECR8315F (15 mg) was incubated with 1.6 mL of 1 mM ethylbenzene in KPi buffer (50 mM pH 7) and 3.1 µL of 3.5% H_2O_2 v/v. The reaction occurred in a 2 mL glass vial placed in a thermo-shaker (Hettich Lab Technology, Netherlands) at 25 °C and 250 rpm. Samples were collected regularly (up to 40 minutes) and analyzed *via* GC-FID analysis and HRP/ABTS assay

to follow the reaction progression. Between one batch and the other, the immobilized enzyme was washed with 1 mL of KPi buffer (50 mM pH 7), vortexed for 2 minutes at maximum speed and centrifuged for 2 minutes at maximum speed. The so washed immobilized enzyme is weighed and moved in a new glass vial, ready for the next round. Samples (1 mL) from the washing step are collected and analyzed *via* Pierce protein assay to assess if any enzyme leaching occurred.

GC analysis

For the qualitative analysis of ethylbenzene, (*R/S*)-1-phenylethanol and acetophenone, a sample containing a final concentration of 25 mM of each chemical was prepared by dissolving a proper amount of the pure organic compounds in dichloromethane (DCM) containing 5 mM of *n*-dodecane as internal standard. The sample was mixed for 2 min and then 300 µL were moved in a glass vial and analyzed on the 2030 Shimadzu GC system equipped with 150 vials auto-sampler and with a flame ionization detector (FID); The column adopted was the CP-Chirasil-Dex CB column (CP7502, NLR0663300, 25 m × 0.25 mm within a coating thickness of 0.25 µm) from Agilent. Method: 70 °C hold 1.0 min, 80 °C/min to 110 °C hold 3.4 min, 25 °C/min to 190 °C hold 21.0 min; inlet temperature: 250 °C; split ratio:1:50 split ratio; helium flow rate: 1 mL/min until 12 minutes, then 3 mL/min until 2 mL/min Retention times: ethylbenzene 3.29 min, acetophenone 5.85 min, *n*-dodecane (I.S.) 6.55 min, (*R*)-1-phenylethanol 9.71 min, (*S*)-1-phenylethanol 10.10 min (see Figure S1).

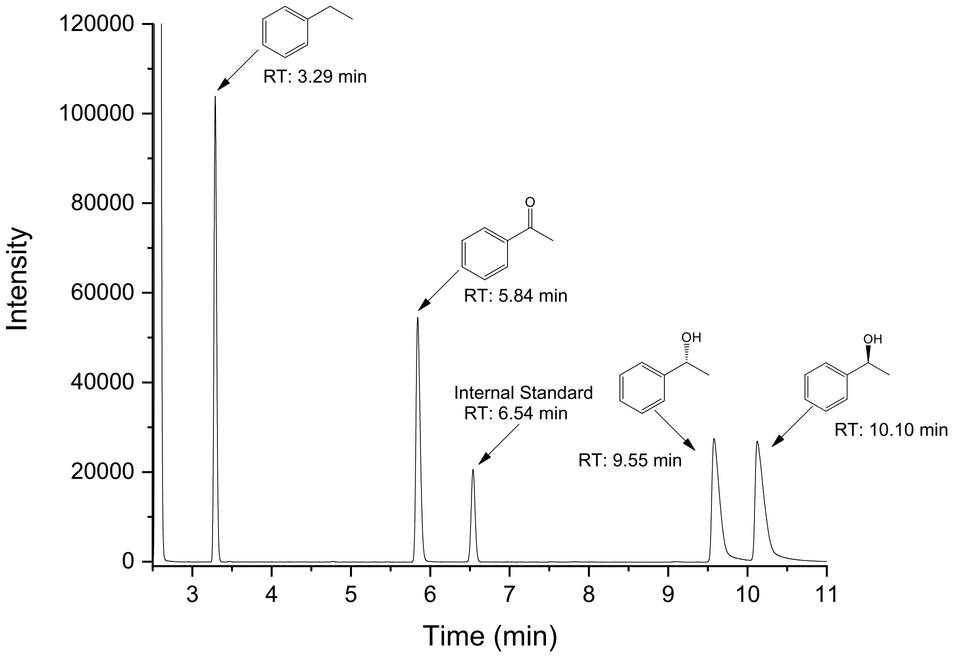

Figure S1. *Retention times of ethylbenzene, n-dodecane, (R)-1-phenylethanol and (S)-1-phenylethanol on GC-FID.*

For the quantitative analysis a multipoint calibration curve was used. For each compound, seven different standard solutions with concentration between 0.39 mM and 25 mM were prepared in DCM (containing 5 mM of *n*-dodecane as internal standard) and analyzed as described before. Since the reaction occurs in aqueous media (KPi buffer 50 mM pH 7) the use of extraction coefficients was mandatory for a proper compound quantification. They were obtained by preparing a standard solution (final concentration 1 mM) for each compound and analyzing them as done before. The extraction coefficients were then calculated as described:

$$(1)\quad \boldsymbol{Extractiom\ coefficient} = \frac{\boldsymbol{Nominal\ concentration(mM)}}{\boldsymbol{Determined\ concentration\ (mM)}}$$

Since not all the experiments were conducted at room temperature the extraction coefficients were determined at different temperature to compensate for the change in solubility related to the temperature variation (see table S4).

Table S4. *Extraction coefficients of ethylbenzene, (R)-1-phenylethanol and acetophenone at different temperature.*

	**17 °C**	**25 °C**	**40 °C**	**60 °C**
**Ethylbenzene**	2.1±0.3	2.5±0.1	2.8±0.1	3.94±0.3
**(*R*)-1-phenylethanol**	1.30±0.02	1.38±0.04	1.35±0.03	1.3832±0.00009
**Acetophenone**	1.47±0.07	1.02±0.02	1.076±0.007	1.14±0.04

For the quantitative analysis of aqueous ethylbenzene hydroxylation reaction, samples (150 µL) were extracted 1:1 with DCM (containing 5 mM of *n*-dodecane as internal standard). To ensure a massive extraction, the samples were incubated with DCM, vortexed for 2 minutes at maximum speed, centrifuged for 2 minutes at maximum speed and then analyzed as described before.

Hydrogen peroxide (H_2O_2) quantification

An ABTS/HRP assay was used for H_2O_2 quantification. It allowed us to follow the course of the ethylbenzene hydroxylation reaction, where the hydrogen peroxide is involved as co-substrate.

For the assay, 1 mL UV cuvette was filled with 900 µL of 1 mM ABTS solution, 50 µL of 0.4 mg/mL horseradish peroxidase (HRP) in KPi buffer (50 mM, pH 7), and 50 µL of the sample collected. The sample was incubated at room temperature for 10 minutes and then its absorbance was measured at 405 nm with a thermostated Cary-60 UV-Vis spectrophotometer. To correlate the absorbance measured to the H_2O_2 concentration a five-points calibration curve was used. Five standard solutions with concentrations between 0.04 and 1 mM were prepared and analyzed as described above (see Figure S2).

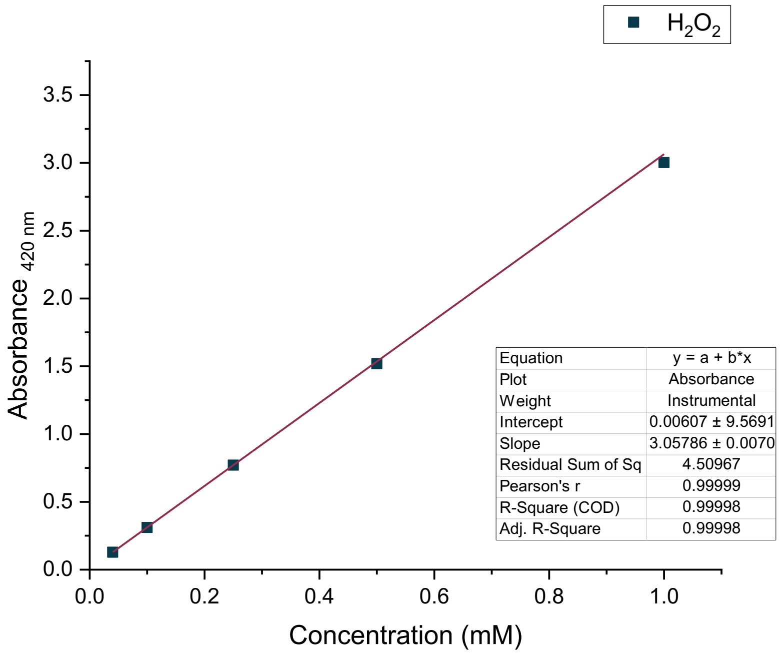

Figure S2. *ABTS/HRP calibration curve.*

RESULTS

Protein purification

Table S5. *Comparison of protein concentration and enzyme activity values of his-tagged PaDa-I and its purified contour part.*

	**Protein concentration ($mg_{protein}$/mL)**	**Enzyme activity**	
		**Volumetric activity (U/mL)**	**Specific activity (U/mg_{Enzyme})**
**His-tagged PaDa-I**	1.4 ± 0.1	442 ± 35	314 ± 25
**Purified his-tagged PaDa-I**	0.77 ± 0.04	484 ± 33	625 ± 23

The specific activity of the purified his-tagged PaDa-I is slightly higher than the non-purified one.

Electrophoresis analysis of protein fractions

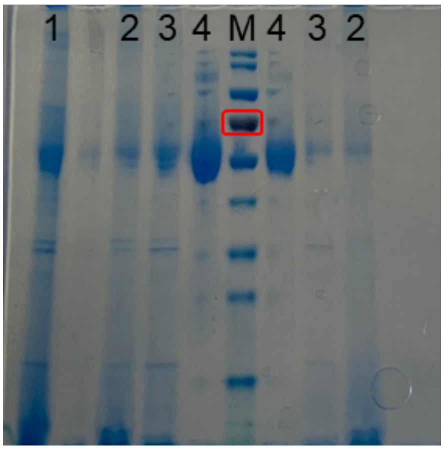

Figure S3. *SDS-PAGE gel obtained after the his-tagged PaDa-I purification. 1) CFE; 2) outflow; 3) wash buffer; 4) purified enzyme; and M) marker ladder.*

Analysis of enzyme-to-carrier ratios

Table S6. *Activity and protein loading of PaDa-I immobilized on the amino carrier ECR8315F according to the enzyme-to-carrier ratios.*

Ratio ($mg_{protein}/g_{carrier}$)	Protein loading ($mg_{protein}/g_{wet\ carrier}$)	Immobilised enzyme activity	
		Based on carrier amount ($U/g_{wet\ carrier}$)	Based on enzyme amount ($U/mg_{Immobilised\ PaDa\text{-}1}$)
0.40	0.27±0.01	6.0±0.7	10±1
0.80	0.62±0.01	16.8±0.2	27.0±0.5
1.00	0.41±0.01	8.0±0.7	20±2
1.50	0.90±0.01	15±14	16±12
2.00	1.51±0.01	18±13	12±9
2.50	1.45±0.01	20±5	13±3
3.00	1.70±0.01	27.5±0.5	16.0±0.3
3.50	1.40±0.01	20.2±0.2	14.0±0.1

Table S7. *Activity and protein loading of the purified PaDa-I immobilized on the Amber EziG^{TM} carrier according to the enzyme-to-carrier ratios.*

Ratio ($mg_{protein}/g_{carrier}$)	Protein loding ($mg_{protein}/g_{wet\ carrier}$)	Immobilization yield (%)	Immobilized enzyme activity		Activity yield (%)
			Based on carrier amount ($U/g_{wet\ carrier}$)	Based on enzyme amount ($U/mg_{Immobilised\ PaDa\text{-}1}$)	
0.6	0.05±0.01	8	9±2	180±45	5
0.8	0.37±0.01	46	12±2	69±13	11
1.0	0.56±0.02	55	34±6	62±12	2
1.5	1.20±0.02	79	21±3	18±2	1
2.0	1.54±0.02	77	42±16	27±10	1

Table S8. *Activity and protein loading of the purified PaDa-I immobilized on the Coral EziG^{TM} carrier according to the enzyme-to-carrier ratios.*

**Ratio ($mg_{protein}/g_{carrier}$)**	**Protein loding ($mg_{protein}/g_{wet\ carrier}$)**	**Immobilization yield (%)**	**Immobilized enzyme activity**		**Activity yield (%)**
			**Based on carrier amount ($U/g_{wet\ carrier}$)**	**Based on enzyme amount ($U/mg_{Immobilised\ PaDa-1}$)**	
0.6	0.218±0.004	22	32±12	158±58	8
0.8	0.46±0.03	56	1.2±0.2	2.6±0.6	0.5
1.0	0.27±0.07	45	14±4	50±15	5
1.5	0.95±0.05	62	27± 3	28±14	1

Table S9. *Activity and protein loading of the purified PaDa-I immobilized on the Opal EziG^{TM} carrier according to the enzyme-to-carrier ratios.*

**Ratio ($mg_{protein}/g_{carrier}$)**	**Protein loding ($mg_{protein}/g_{wet\ carrier}$)**	**Immobilization yield (%)**	**Immobilized enzyme activity**		**Activity yield (%)**
			**Based on carrier amount ($U/g_{wet\ carrier}$)**	**Based on enzyme amount ($U/mg_{Immobilised\ PaDa-1}$)**	
0.8	0.398±0.002	50	189±6	475±15	9
1.0	0.5195±0.0007	52	166±10	319±19	28
1.2	0.764±0.004	92	47±7	67±9	55
1.5	1.388±0.002	97	69±34	50±25	8
2.0	1.357±0.002	97	20±6	15±5	6

Analysis of enzyme half-life time determination

After measuring the activity of both the free and the immobilized PaDa-I for one month, the deactivation constant (k_d) is obtained by plotting the measured activity (U mg^{-1}) against time (day). The half-life time is then calculated according to Equation 2.

$$(2)\quad t_{½} = \frac{ln(2)}{k_d}$$

Table S10. *Determination of the half-life time of both free AaeUPO PaDa-I and covalently immobilized enzyme on Lifetech™ ECR8315F.*

Enzyme	k_d (days)	$t_{½}$ (h)
Free PaDa-I	0.3124	53
Immobilized PaDa-I	0.02007	829

Enzyme stability experiment

No significant enzyme deactivation occurred when the immobilized PaDa-I was incubated in ethylbenzene, (*R*)-1-phenylethanol or acetophenone at room temperature for 120 hours.

Enzyme leaching experiment

A negligible enzyme leaching was determined when the immobilized PaDa-I was incubated in ethylbenzene, (*R*)-1-phenylethanol or acetophenone at room temperature for 120 hours.

Progress curves

Three different experiments were conducted to optimize the reaction condition for the hydroxy functionalization of 0.0016 mmol of ethylbenzene. Firstly, three different amounts of covalently immobilized PaDa-I were evaluated the in order to determine the optimum enzyme amount needed (Figure S4).

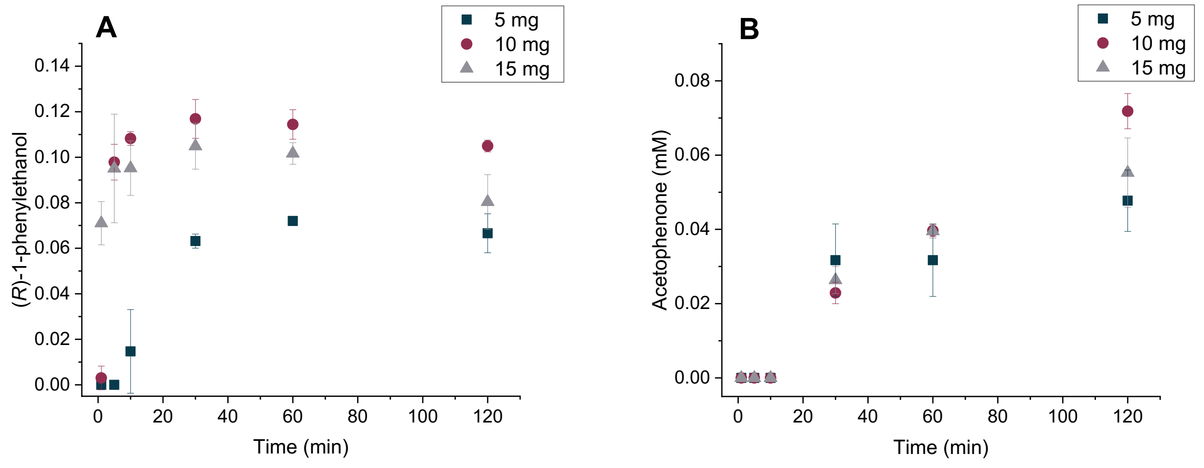

Figure S4. *Progress curves with different amounts of immobilized enzyme; product formation (A) and overoxidized product (B).*

Once 10 mg of immobilized enzyme had been determined as the ideal amount to use the hydroxy functionalization of 0.0016 mmol of ethylbenzene, different shaking intensity were analyzed (see Figure 5). The performed experiment proved that the highest product formation is reached when the samples are incubated at 250 rpm.

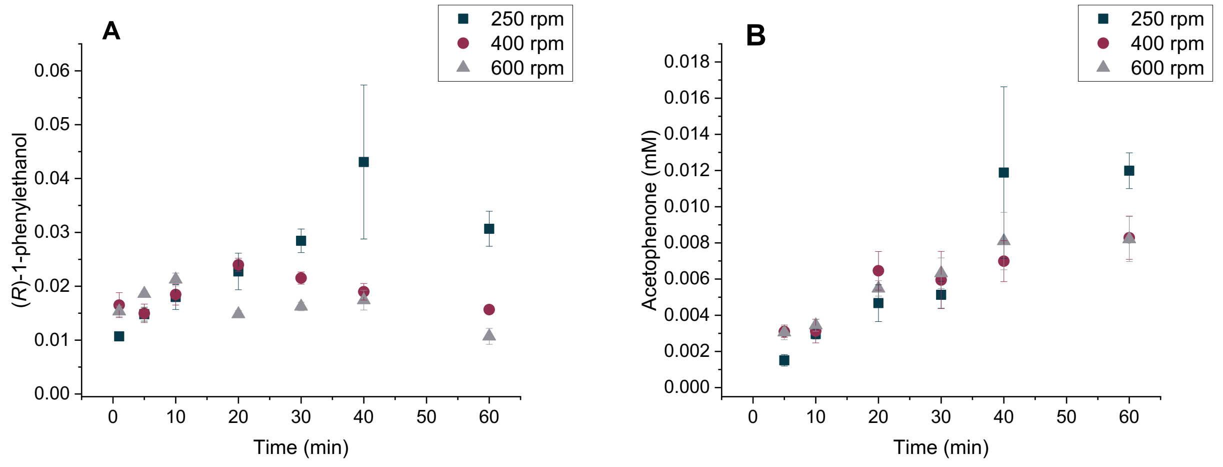

Figure S5. *Progress curves with different intensity of shaking; product formation (A) and overoxidized product (B).*

Eventually, the effect of the temperature on the reaction was studied; 10 mg of immobilized enzyme were incubated at 250 rpm and four different temperatures were applied (Figure S6).

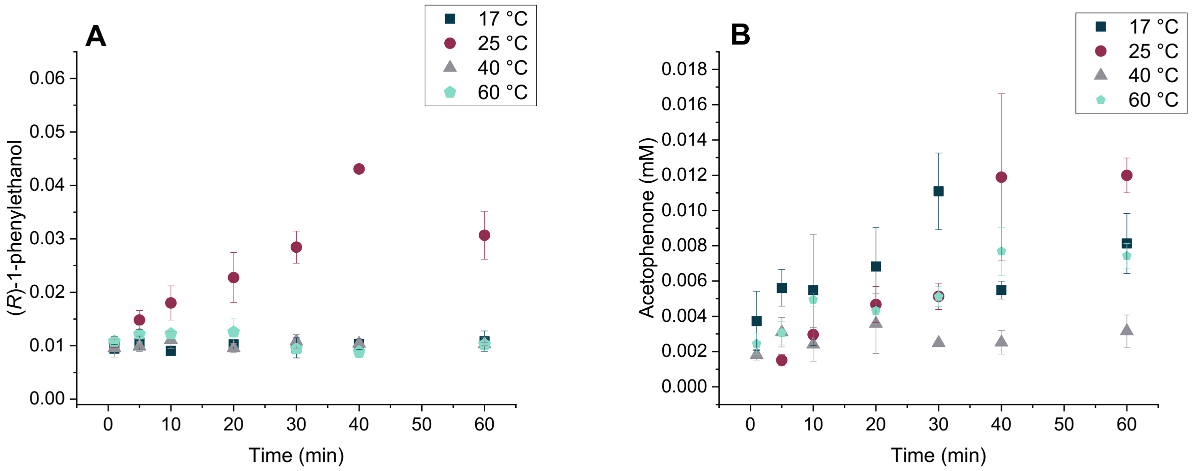

Figure S6. *Progress curves at different temperatures; product formation (A) and overoxidized product (B).*

Repetitive batch experiments

Once the optimum reaction conditions were identified thanks to the progress curve experiments, the reusability of the immobilized enzyme was evaluated and, as proved with the repetitive batch experiments, the immobilized enzyme can be reused up to seven times. However, the decrease in products concentration, detected *via* GC analysis, as well as the reduction in H_2O_2 consumption, determined *via* ABTS/HRP assay, clearly demonstrate the decline of enzyme activity between one batch and the other.

REFERENCES

Cassimjee, K. E. and H.-J. Federsel (2018). EziG: An Universal Platform for Immobilise Enzyme, Royal Society of Chemistry.

Hobisch, M. and S. Kara (2021). "Automated Feeding and Fermentation Phase Transitions for the Production of Unspecific Peroxygenase by Pichia pastoris."

Purolite Life Sciences. (2015). "Lifetech™ ECR Enzyme Immobilization Procedures." Retrieved 29-08-2020, 2020, from https://www.puro-lite.com/dam/jcr:3c5b978b-3829-4f92-8654-390863042f09/Lifetech%20ECR%20immobi-lisation%20procedures.pdf.

Thermo Fisher Scientific (2015). Ni-Nta purification system, Life Technologies Corporation.
